# Dietary fiber density and incident gross motor limitation among middle-aged and older adults: a prospective cohort analysis

**DOI:** 10.3389/fnut.2026.1922715

**Published:** 2026-08-28

**Authors:** Tao Cai, Zhongxing Liu, Yunsong Zhang, Yating Li, Qi Liu, Qi Zhang

**Affiliations:** 1 School of Sports Medicine and Health, Chengdu Sport University, Chengdu, China; 2 Sports Medicine Key Laboratory of Sichuan Province, Chengdu, China; 3 Affiliated Sports Hospital of Chengdu Sport University, Chengdu, China; 4 Institute of Sports Medicine and Health, Chengdu Sport University, Chengdu, China; 5 Traditional Chinese Medicine Prevention and Treatment Center, Chengdu Integrated TCM and Western Medicine Hospital, Chengdu, China; 6 Hejiang Hospital of Traditional Chinese Medicine, Luzhou, China; 7 Hejiang Hospital of Traditional Chinese Medicine Affiliated to Southwest Medical University, Luzhou, China

**Keywords:** ageing, dietary fiber, fiber density, gross motor limitation, health and retirement study, nutrition epidemiology, physical function, prospective cohort

## Abstract

**Introduction:**

Preserving physical function is a central goal of ageing research, yet the dietary factors that precede early motor limitation remain incompletely defined.

**Methods:**

Here we examined whether dietary fiber density was associated with incident gross motor limitation in the 2013 Health Care and Nutrition Study linked to the Health and Retirement Study. Among 5,388 adults aged 50 years or older who were free of gross motor limitation at baseline, 2,018 developed incident limitation during biennial follow-up through 2022.

**Results:**

In the fully adjusted modified Poisson model, each 1-s.d. increment in fiber density was associated with a risk ratio of 0.86 (95% CI, 0.82 to 0.90), and the highest versus lowest quartile had a risk ratio of 0.72 (95% CI, 0.64 to 0.81). Augmented inverse probability weighting estimated a 9.0 percentage-point lower absolute risk in the highest versus lowest quartile. Estimates were unchanged after multiple imputation for missing covariates. In Fine-Gray models treating death as a competing event, the subdistribution hazard ratio was 0.82 per 1-s.d. increment (95% CI, 0.77 to 0.87). Results were also consistent across alternative exposure and outcome definitions, interval-aware models, diet-pattern adjustment and secondary functional outcomes.

**Conclusion:**

Higher fiber density was consistently associated with lower functional-limitation risk, but it may index a broader constellation of dietary and lifestyle factors; causal effects cannot be inferred.

## Introduction

Preserving mobility and functional independence is a central goal of aging research. Functional limitation, disability, frailty and comorbidity are related but distinct states, and the transition from early motor difficulty to disability often marks a clinically meaningful loss of reserve in later life (1, 2). Measures of lower-extremity and gross motor performance predict subsequent disability, institutionalization and mortality, underscoring that mobility decline is not merely a symptom of aging but a pathway through which aging becomes disabling (3–8). Identifying modifiable factors that precede gross motor limitation may therefore help shift prevention earlier, before limitations become established.

Diet is one such modifiable factor, but its relationship with functional aging remains incompletely defined. Higher-quality dietary patterns, including Mediterranean-style diets, have been associated with lower risks of frailty and pre-frailty in older adults (9–12). Dietary fiber is a particularly plausible component of this relationship because it captures carbohydrate quality, food structure and plant-food density more directly than total carbohydrate intake alone. In large systematic reviews, higher fiber intake and whole-grain consumption have been linked to lower risks of cardiovascular disease, type 2 diabetes, colorectal cancer and mortality (13–16). Prospective evidence has also connected carbohydrate quality, including fiber intake, with multidomain successful aging (17). Yet these studies have generally emphasized chronic disease endpoints, global aging composites or broad diet patterns rather than incident gross motor limitation as a specific functional outcome.

Biologically, dietary fiber may influence functional aging through several intersecting pathways. Fermentable fiber shapes gut microbial ecology and provides substrate for short-chain fatty acid production, which has been implicated in host metabolic, immune and inflammatory regulation (18–20). Studies of older adults further suggest that diet, gut microbiota and inflammatory aging are linked to frailty and health status (21–23). These pathways are relevant to muscle function, sarcopenia-related vulnerability and physical resilience (24), but epidemiological evidence linking fiber density to later-life motor limitation remains sparse. This distinction matters because fiber intake is strongly correlated with overall diet quality, physical activity, body weight and baseline health; analyses of functional outcomes therefore require careful adjustment, sensitivity analyses and attention to reverse causation.

Here, we used the 2013 Health Care and Nutrition Study linked to the nationally representative Health and Retirement Study to examine whether dietary fiber density was associated with incident gross motor limitation among middle-aged and older US adults (25). We focused on fiber density, expressed as grams per 1,000 kcal, to capture fiber exposure relative to total energy intake (26). Among participants free of gross motor limitation at baseline, we evaluated subsequent limitation across biennial follow-up waves through 2022. The analysis combined survey-weighted models, absolute-risk estimation, dose–response analyses, interval-aware sensitivity analyses and outcome-specific secondary analyses of ADL disability, IADL disability and mobility limitation. This design examines whether fiber density is a consistent dietary signal for functional aging, while preserving the observational boundary that the findings cannot by themselves establish causality.

## Methods

### Study design and population

We conducted a prospective cohort analysis using the 2013 Health Care and Nutrition Study (HCNS), linked to RAND Health and Retirement Study (HRS) longitudinal data through 2022 (25, 27). Baseline functional status was assessed at HRS wave 11 in 2012, dietary exposure was measured in HCNS 2013, and incident gross motor limitation was ascertained from HRS waves 12–16, covering follow-up from 2014 through 2022.

The analytic cohort was restricted to participants aged 50 years or older who had a positive HCNS nutrition weight, non-missing dietary fiber density, no gross motor limitation at baseline and observed follow-up outcome status. Of 8,035 HCNS-linked participants, 7,608 were aged 50 years or older, 7,606 had known baseline gross motor status, 5,810 were free of gross motor limitation at baseline, 5,656 also had a positive nutrition weight and non-missing fiber density, and 5,504 had observed follow-up outcome status. Of these, 5,388 had complete Model 3 covariate data and comprised the primary complete-case cohort. Thus, 152 participants were excluded because follow-up outcome status was unavailable and 116 (2.1% of the outcome-known eligible cohort) because at least one Model 3 covariate was missing (Supplementary Figure 1, Supplementary Table 1).

### Dietary fiber density

HCNS dietary intake was assessed using a mailed, self-administered semiquantitative food-frequency questionnaire based on the Harvard food-frequency questionnaire. Participants reported their average intake during the previous year for 164 prespecified foods and beverages, using item-specific portion sizes and frequency categories, and could report up to three additional foods. HRS converted categorical responses to servings per day and derived 215 nutrient variables using Harvard nutrient composition tables. In the final Version 5.0 nutrient file, respondents with extremely sparse dietary data were excluded, and missing food-item responses were imputed by HRS using age, sex, race, education, body mass index and six sentinel foods; 97% of respondents completed at least 90% of food items (27). The parent Harvard semiquantitative food-frequency questionnaire has demonstrated validity and reproducibility for foods and food groups, although the precise HCNS adaptation has not, to our knowledge, undergone a separate validation study (28).

The primary exposure was dietary fiber density, calculated from the HCNS-derived variables as total dietary fiber intake divided by total energy intake and expressed as grams per 1,000 kcal. Fiber density was selected because it captures the fiber content of the diet relative to energy intake rather than total fiber intake alone (26). Because the released nutrient totals incorporated the HCNS item-level missing-data procedure, the analytic exposure had no missing values among HCNS-linked participants, among those entering the incident-risk set or among the final complete-case sample (Supplementary Table 1). We analyzed fiber density as a standardized continuous variable and as quartiles. In the complete-case sample, quartile cut points were 1.53, 8.58, 10.59, 12.88 and 31.60 g per 1,000 kcal.

Exposure-definition sensitivity analyses examined total fiber intake in grams per day, energy-residual fiber, fiber density without total energy adjustment, winsorized fiber density, exclusion of extreme energy reports and exclusion of extreme fiber-density values (Supplementary Table 2).

### Ascertainment of incident gross motor limitation

The primary outcome was incident gross motor limitation during follow-up. Gross motor limitation was defined using RAND HRS gross motor limitation counts (r11grossa–r16grossa), which range from 0 to 5 and summarize reported difficulty with walking one block, walking across a room, climbing one flight of stairs, getting in or out of bed and bathing. These tasks map onto established disablement and lower-extremity function frameworks (1, 3). Participants with a non-zero gross motor limitation count at wave 11 were excluded from the primary analysis. Among participants with a count of zero at wave 11, incident limitation was defined as a count of one or more at any follow-up wave from wave 12 through wave 16.

We evaluated three stricter outcome definitions in sensitivity analyses (Supplementary Table 3): at least two gross motor limitation items in any follow-up wave, at least three items in any follow-up wave, and persistent or recurrent limitation, defined as at least one gross motor limitation in at least two follow-up waves. A composite endpoint of incident gross motor limitation or death was retained as an additional sensitivity analysis; death was also modeled as a distinct competing event in the Fine-Gray analyses described below.

### Covariates

The fully adjusted model included demographic, socioeconomic, lifestyle, anthropometric, cardiometabolic and baseline health covariates. These were age, sex, race or ethnicity, education, total energy intake, body mass index, current smoking, alcohol use, weekly moderate or vigorous physical activity, hypertension, diabetes and self-rated health. Continuous covariates were standardized before modeling. HCNS nutrition weights were used in the weighted analyses.

We estimated three nested models. Model 1 adjusted for age, sex, race or ethnicity, education and total energy intake. Model 2 additionally adjusted for body mass index, current smoking, alcohol use and physical activity. Model 3, the primary model, additionally adjusted for hypertension, diabetes and self-rated health (Supplementary Table 4).

## Statistical analysis

Baseline characteristics were summarized across quartiles of dietary fiber density using weighted means with standard deviations or weighted percentages, as appropriate. Standardized mean differences compared the highest and lowest quartiles (Table 1).

**Table 1:** Baseline characteristics by quartile of dietary fiber density.

Characteristic	Summary	Q1	Q2	Q3	Q4	SMD, Q4 vs. Q1
Unweighted sample size	*n*	1,347	1,347	1,347	1,347	
Dietary fiber density, g/1000 kcal	Weighted mean (SD)	7.0 (1.2)	9.6 (0.6)	11.6 (0.7)	15.6 (2.5)	4.39
Age, years	Weighted mean (SD)	63.1 (8.7)	65.3 (9.4)	65.0 (9.3)	65.7 (9.6)	0.29
Education, years	Weighted mean (SD)	13.1 (2.3)	13.7 (2.5)	13.9 (2.7)	13.7 (3.1)	0.21
Total energy intake, kcal/day	Weighted mean (SD)	2003.7 (1150.8)	1863.2 (953.6)	1790.6 (989.9)	1733.8 (730.1)	0.28
BMI, kg/m^2^	Weighted mean (SD)	28.3 (5.5)	28.5 (5.4)	28.1 (5.5)	27.3 (5.2)	0.18
Self-rated health score	Weighted mean (SD)	2.6 (0.9)	2.5 (0.9)	2.4 (0.9)	2.3 (1.0)	0.34
Baseline non-gross functional burden	Weighted mean (SD)	1.5 (1.9)	1.4 (1.9)	1.2 (1.7)	1.1 (1.6)	0.25
Female: yes	Weighted %	39.9	50.1	56.2	63.5	0.49
Current smoking: yes	Weighted %	26.0	12.7	6.8	4.6	0.62
Any drinking: yes	Weighted %	65.2	64.7	63.3	58.1	0.15
Moderate/vigorous physical activity weekly: yes	Weighted %	73.3	79.6	83.3	87.3	0.36
Hypertension: yes	Weighted %	53.1	51.0	50.5	46.1	0.14
Diabetes: yes	Weighted %	13.8	15.1	18.8	19.8	0.16
Race/ethnicity: White/Caucasian	weighted %	83.3	85.9	87.5	83.3	0.00
Race/ethnicity: Black/African American	Weighted %	11.8	9.2	7.2	7.8	0.13
Race/ethnicity: other	Weighted %	5.0	4.9	5.3	8.8	0.15

The primary association between fiber density and incident gross motor limitation was estimated using survey-weighted logistic regression. Because incident gross motor limitation was common, modified Poisson models were used to estimate risk ratios for the main interpretation (29). Absolute-risk contrasts comparing the highest and lowest quartiles were estimated using augmented inverse probability weighting (AIPW), with bootstrap confidence intervals (30).

Dose–response patterns were examined using survey-weighted natural spline models with 4 degrees of freedom. Adjusted marginal risks and parametric 95% confidence intervals were generated from multivariate-normal coefficient draws, with the supporting spline diagnostic shown in Supplementary Figure 2. Time-to-event sensitivity analyses used discrete-time hazard models across biennial follow-up intervals. Approximate survey-weighted Cox models were also fitted using the first wave at which gross motor limitation was reported as the event time. These Cox models were considered sensitivity analyses because exact onset dates were not observed between biennial HRS interviews (Supplementary Table 5).

Robustness analyses examined diet-pattern adjustment, health selection, complete-case selection, competing death, alternative outcome definitions, alternative exposure definitions and subgroup heterogeneity. Diet-pattern analyses additionally adjusted for broad diet-quality, unfavorable-diet, carbohydrate-quality and plant-food-quality modules (Supplementary Table 6). Health-selection analyses excluded participants with major chronic disease, baseline non-gross functional burden, poor baseline health or early follow-up events. Complete-case selection was assessed using inverse probability weighting (Supplementary Table 7). Subgroup analyses evaluated heterogeneity by age, sex, obesity status, physical activity, diabetes, hypertension, self-rated health and major chronic disease, with false-discovery-rate adjustment for multiple subgroup tests (Supplementary Table 8). *E*-values were calculated as descriptive benchmarks for unmeasured confounding (Supplementary Table 9) (31). Secondary functional-outcome analyses evaluated incident ADL disability, IADL disability and mobility limitation with the same fully adjusted covariate structure (Supplementary Table 10).

Primary analyses used complete cases. We tabulated missingness for each Model 3 covariate among the 5,504 eligible participants with observed outcome status. As a sensitivity analysis, missing Model 3 covariates were imputed using multiple imputation by chained equations (20 imputations; 10 iterations). The imputation model included the outcome, fiber density, the HCNS nutrition weight and all Model 3 covariates; predictive mean matching was used for continuous or ordinal variables, logistic regression for binary variables and multinomial logistic regression for race or ethnicity. Exposure and outcome values were not imputed. Survey-weighted estimates were pooled using Rubin’s rules (32).

To distinguish incident limitation from death, we fitted HCNS-weighted Fine-Gray subdistribution hazard models with robust standard errors clustered by participant (33). The first observed limitation wave was the event time, death before observed limitation was the competing event, and participants with neither outcome were censored at their last observed limitation-free wave. These models were treated as approximate sensitivity analyses because event timing was assigned to biennial interview waves.

Statistical tests were two-sided, and *p* < 0.05 was considered significant. Analyses were performed with R version 4.6.0.

## Results

### Study population

The primary complete-case cohort included 5,388 participants free of gross motor limitation at baseline, of whom 2,018 developed incident gross motor limitation; the crude weighted event rate was 32.7%. Among the 5,504 outcome-known eligible participants, 116 (2.1%) had at least one missing Model 3 covariate. Per-variable missingness ranged from 0 to 1.0% (Supplementary Table 1).

Baseline characteristics varied across quartiles of dietary fiber density (Table 1). Compared with participants in the lowest quartile, those in the highest quartile had lower smoking prevalence (4.6% versus 26.0%), more frequent weekly moderate or vigorous physical activity (87.3% versus 73.3%), better self-rated health and lower baseline non-gross functional burden. Participants in the highest quartile were also more likely to be women (63.5% versus 39.9%). These imbalances supported the use of multivariable adjustment and extensive sensitivity analyses.

### Dietary fiber density and incident gross motor limitation

Higher dietary fiber density was associated with lower risk of incident gross motor limitation in the fully adjusted model (Table 2, Supplementary Table 4). Per 1-s.d. increment in fiber density, the survey-weighted logistic odds ratio was 0.77 (95% confidence interval (CI), 0.71–0.84; *p* < 0.001). When fiber density was modeled in quartiles, participants in the highest quartile had lower odds of incident gross motor limitation than those in the lowest quartile (odds ratio, 0.55; 95% CI, 0.44–0.68; *p* < 0.001).

**Table 2:** Core associations between dietary fiber density and incident gross motor limitation.

Analysis	Contrast	Measure	*N*	Events	Estimate	*p*
Primary logistic model	Per SD	OR	5,388	2018	0.77 (0.71–0.84)	<0.001
Primary logistic model	Q4 vs. Q1	OR	5,388	2018	0.55 (0.44–0.68)	<0.001
Modified Poisson model	Per SD	RR	5,388	2018	0.86 (0.82–0.90)	<0.001
Modified Poisson model	Q4 vs. Q1	RR	5,388	2018	0.72 (0.64–0.81)	<0.001
AIPW absolute-effect model	Q4 vs. Q1	Risk difference, pp	2,694	1,016	−9.0 (−13.0 to −4.6)	Bootstrap CI
AIPW absolute-effect model	Q4 vs. Q1	RR	2,694	1,016	0.76 (0.67–0.87)	Bootstrap CI
Discrete-time hazard RR, per SD	Per SD	Hazard RR	5,313 participants	1923	0.83 (0.78–0.88)	<0.001
Approximate Cox HR, Q4 vs. Q1	Q4 vs. Q1	HR	5,388	2018	0.60 (0.51–0.70)	<0.001
Approximate Cox HR, per SD	Per SD	HR	5,388	2018	0.81 (0.76–0.86)	<0.001
OR after broad diet-quality adjustment	Per SD	OR	5,388	2018	0.85 (0.76–0.96)	0.006

Because the outcome was frequent, risk ratios were used as the main relative effect scale. In the modified Poisson model, each 1-s.d. increment in fiber density was associated with a risk ratio of 0.86 (95% CI, 0.82–0.90; *p* < 0.001), and the risk ratio comparing the highest with the lowest quartile was 0.72 (95% CI, 0.64–0.81; *p* < 0.001).

**Figure 1:**
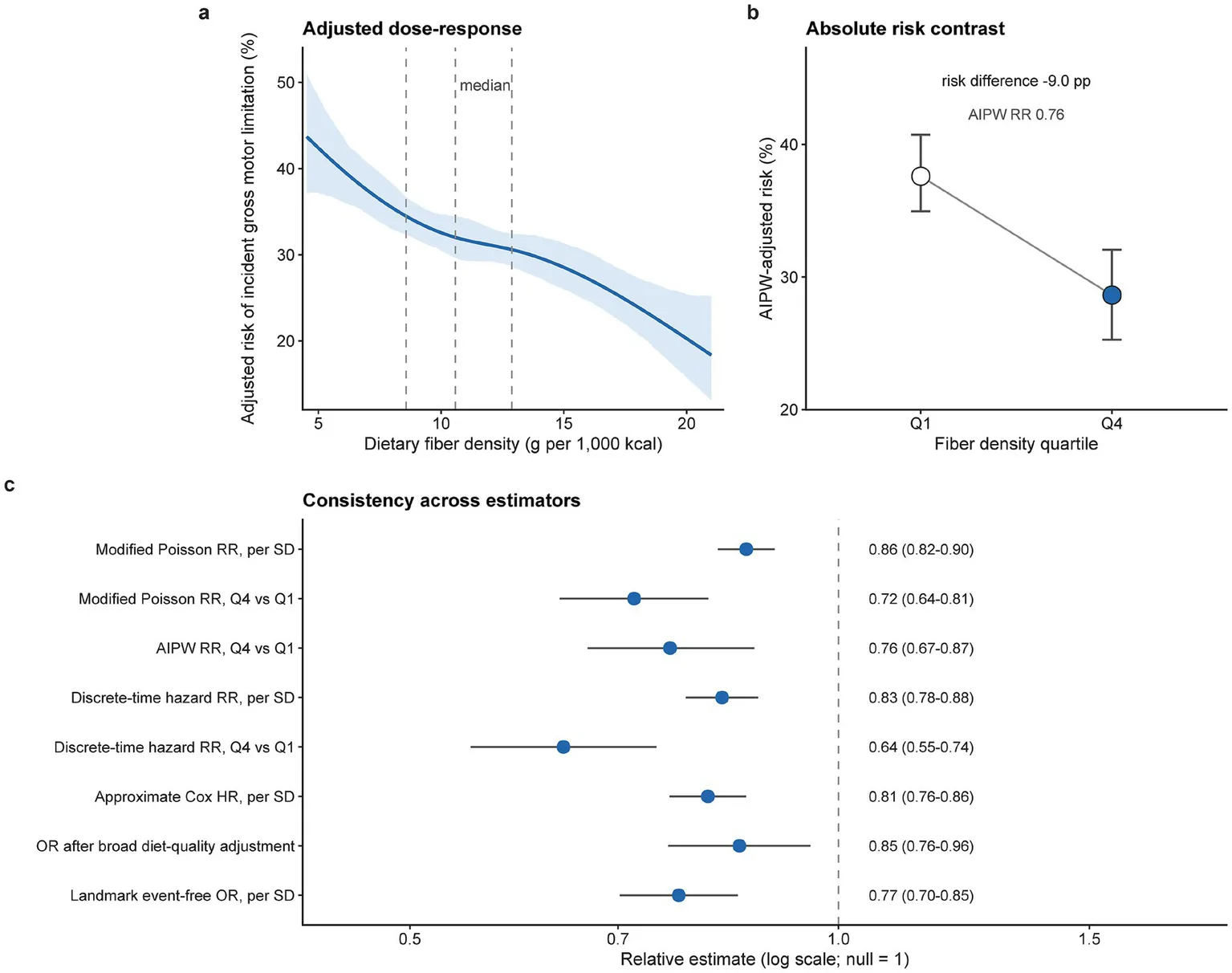
Dietary fiber density and incident gross motor limitation. **(a)** Adjusted marginal risk of incident gross motor limitation across dietary fiber density, estimated using a survey-weighted natural spline model. Shading denotes a parametric 95% confidence interval, and dashed vertical lines denote the 25th, 50th and 75th percentiles. **(b)** AIPW-adjusted absolute risk comparing the lowest and highest quartiles of dietary fiber density. Points indicate estimated risks and vertical bars indicate 95% confidence intervals. **(c)** Key relative estimates across primary and sensitivity models. Points and horizontal bars indicate point estimates and 95% confidence intervals. RR, risk ratio; HR, hazard ratio; OR, odds ratio; AIPW, augmented inverse probability weighting.

Absolute-risk estimation gave a similar interpretation (Figure 1b, Table 2). In the AIPW analysis comparing the highest and lowest quartiles, the estimated risk of incident gross motor limitation was 37.6% in the lowest quartile and 28.6% in the highest quartile. This corresponded to a risk difference of −9.0 percentage points (95% CI, −13.0 to −4.6) and an AIPW risk ratio of 0.76 (95% CI, 0.67–0.87).

### Dose–response and interval-aware analyses

Spline-based marginal risk curves showed a graded inverse association across the central exposure range (Figure 1a, Supplementary Figure 2). The median fiber density was 10.59 g per 1,000 kcal; the 25th and 75th percentiles were 8.58 and 12.88 g per 1,000 kcal. The dose–response pattern was not limited to an extreme contrast between the lowest and highest quartiles.

Time-to-event sensitivity analyses supported the primary findings (Figure 1c, Table 2, Supplementary Table 5). In discrete-time hazard models, the hazard risk ratio was 0.83 per 1-s.d. increment in fiber density (95% CI, 0.78–0.88; *p* < 0.001). These models included 17,623 person-periods from 5,313 participants and 1,923 events.

Approximate survey-weighted Cox models yielded consistent estimates. In the main complete-case set, the hazard ratio was 0.81 per 1-s.d. increment (95% CI, 0.76–0.86; *p* < 0.001) and 0.60 for the highest versus lowest quartile (95% CI, 0.51–0.70; *p* < 0.001). These estimates are interpreted as sensitivity analyses because gross motor limitation onset was observed at interview waves rather than exact dates.

### Robustness analyses

Multiple-imputation analyses included all 5,504 outcome-known eligible participants (2,057 events) and closely reproduced the complete-case findings. Per 1-s.d. increment, the pooled odds ratio was 0.77 (95% CI, 0.71–0.84; *p* < 0.001) and the pooled risk ratio was 0.86 (95% CI, 0.82–0.90; *p* < 0.001). For the highest versus lowest quartile, the corresponding estimates were 0.54 (95% CI, 0.44–0.67; *p* < 0.001) and 0.72 (95% CI, 0.64–0.81; *p* < 0.001), respectively (Supplementary Table 11).

The association was stable across alternative outcome definitions (Supplementary Table 3). Per 1-s.d. increment in fiber density, the odds ratio was 0.84 (95% CI, 0.76–0.93; *p* < 0.001) for incident limitation defined as at least two gross motor items, 0.80 (95% CI, 0.69–0.93; *p* = 0.003) for at least three items, and 0.83 (95% CI, 0.74–0.92; *p* < 0.001) for persistent or recurrent limitation. Alternative exposure definitions, including total fiber, energy-residual fiber, winsorized fiber density and outlier exclusions, also yielded inverse estimates (Supplementary Table 2).

Adjustment for broader dietary patterns attenuated but did not eliminate the association. After additional adjustment for a broad diet-quality module, the odds ratio per 1-s.d. increment in fiber density was 0.85 (95% CI, 0.76 to 0.96; *p* = 0.006; Figure 1c, Table 2, Supplementary Table 6). Models additionally accounting for unfavorable-diet, carbohydrate-quality and plant-food-quality modules produced directionally consistent estimates.

Analyses addressing selection and reverse causation gave similar results. Complete-case selection weighting did not materially change the primary estimate (Supplementary Table 7). The inverse association also persisted after excluding participants with major chronic disease, excluding those with baseline non-gross functional burden, restricting to the healthiest subset and applying a 2014 landmark event-free analysis.

Treating death as a competing event rather than combining it with functional limitation yielded consistent estimates. In the Fine-Gray analysis of 5,388 participants, 2,018 experienced gross motor limitation, 514 died before observed limitation and 2,856 were censored. The subdistribution hazard ratio was 0.82 per 1-s.d. increment in fiber density (95% CI, 0.77–0.87; *p* < 0.001) and 0.62 for the highest versus lowest quartile (95% CI, 0.53–0.73; *p* < 0.001; Supplementary Table 11). In the additional composite-endpoint analysis of incident limitation or death, the odds ratio per 1-s.d. increment was 0.76 (95% CI, 0.70–0.82; *p* < 0.001) and the modified Poisson risk ratio was 0.88 (95% CI, 0.85–0.91; *p* < 0.001; Supplementary Table 5).

Secondary functional-outcome analyses were directionally consistent, with odds ratios per 1-s.d. increment of 0.89 (95% CI, 0.82–0.97; *p* = 0.006) for incident ADL disability, 0.88 (95% CI, 0.81–0.96; *p* = 0.003) for incident IADL disability and 0.87 (95% CI, 0.79–0.96; *p* = 0.004) for incident mobility limitation (Supplementary Table 10).

Across the integrated robustness atlas, all 55 odds-ratio specifications were inverse, and 54 had 95% CIs excluding the null. All 25 risk-ratio specifications were inverse and excluded the null. Hazard-ratio, hazard-odds-ratio and hazard-risk-ratio specifications were also consistently inverse and excluded the null. Full relative and absolute robustness atlases are provided in Supplementary Figures 3, 4. *E*-value analyses were retained as descriptive unmeasured-confounding benchmarks (Supplementary Table 9).

### Exploratory subgroup analyses

**Figure 2:**
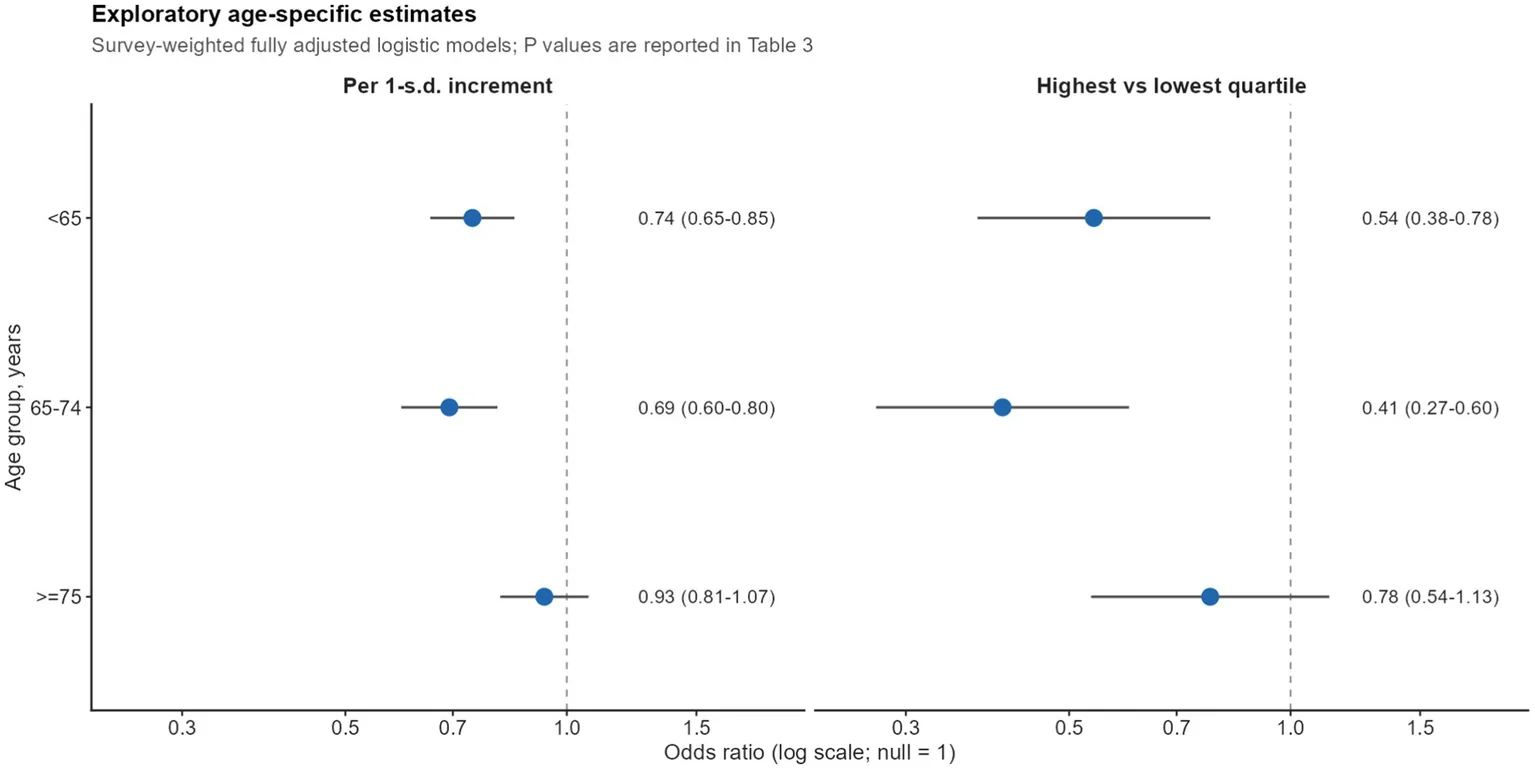
Exploratory age-specific estimates. Age-specific fully adjusted odds ratios are shown for dietary fiber density modeled per 1-s.d. increment and as the highest versus lowest quartile. Points and horizontal bars indicate odds ratios and 95% confidence intervals. This figure should be treated as exploratory; age was the clearest heterogeneity signal, but subgroup findings should not be overinterpreted as a biological threshold.

Age showed the clearest heterogeneity signal (Figure 2, Table 3, Supplementary Figure 5, Supplementary Table 8). In the modified Poisson interaction model, the age-group interaction *p* value was 1.52 × 10^−5^, with a false-discovery-rate adjusted *p* value of 1.21 × 10^−4^. The logistic interaction was weaker after false-discovery-rate adjustment (*p* = 0.0079; adjusted *p* = 0.063). In fully adjusted age-specific logistic models, the odds ratios per 1-s.d. increment were 0.74 (95% CI, 0.65–0.85) among participants younger than 65 years, 0.69 (95% CI, 0.60–0.80) among those aged 65–74 years and 0.93 (95% CI, 0.81–1.07) among those aged 75 years or older. Sex-stratified estimates were similar in women (odds ratio, 0.78; 95% CI, 0.70–0.86; risk ratio, 0.86; 95% CI, 0.82–0.91) and men (odds ratio, 0.77; 95% CI, 0.67–0.88; risk ratio, 0.84; 95% CI, 0.78–0.92), with no evidence of interaction (logistic *p* = 0.559; modified Poisson *p* = 0.281; Supplementary Table 8). Other subgroup variables did not show comparable false-discovery-rate adjusted evidence of interaction.

**Table 3:** Exploratory age-specific estimates.

Age group	Contrast	*N*	Events	OR (95% CI)	*p*
<65	per SD	2,485	666	0.74 (0.65–0.85)	<0.001
65–74	per SD	1,576	622	0.69 (0.60–0.80)	<0.001
≥75	per SD	1,327	730	0.93 (0.81–1.07)	0.319
<65	Q4 vs. Q1	2,485	666	0.54 (0.38–0.78)	<0.001
65–74	Q4 vs. Q1	1,576	622	0.41 (0.27–0.60)	<0.001
≥75	Q4 vs. Q1	1,327	730	0.78 (0.54–1.13)	0.186

## Discussion

In this prospective analysis of HRS-HCNS participants free of gross motor limitation at baseline, higher dietary fiber density was associated with a lower subsequent risk of incident gross motor limitation. The finding was not restricted to a single modeling choice: inverse associations were observed on odds-ratio, risk-ratio, absolute-risk and interval-aware time-to-event scales and were robust to multiple imputation and Fine-Gray competing-risk analysis. Secondary analyses of incident ADL disability, IADL disability and mobility limitation were directionally consistent. The adjusted 9.0 percentage-point contrast between the highest and lowest fiber-density quartiles is a population-level association, however, and should not be interpreted as an expected treatment effect or number needed to treat.

These findings are relevant because gross motor limitation can precede more severe disability. Functional limitation, frailty, comorbidity and disability are related but separable states, and early motor difficulty may represent a window in which prevention remains plausible before dependence becomes established (1–4). In that context, the consistency across gross motor limitation and broader functional outcomes supports the interpretation that fiber density may mark a diet-related dimension of functional resilience rather than only a disease-specific risk marker.

Our results extend prior work on carbohydrate quality, diet quality and healthy aging. Higher dietary fiber intake has been associated with lower risks of cardiometabolic disease, type 2 diabetes, colorectal cancer and mortality in large systematic reviews and prospective cohorts (13–16). Carbohydrate quality, including fiber intake, has also been linked to multidomain successful aging (17). Evidence from dietary-pattern research suggests that healthier diets, particularly Mediterranean-style patterns, are associated with lower frailty risk in older adults (9–11). The present study adds a more specific functional endpoint by focusing on incident gross motor limitation in a large longitudinal aging cohort, while using fiber density rather than total fiber intake to account for differences in total energy intake (25, 26).

Several mechanisms could plausibly connect fiber-rich diets with preserved physical function, although this study was not designed to test them directly. Fermentable fibers alter gut microbial ecology and support production of short-chain fatty acids, which have been implicated in immune, inflammatory and metabolic regulation (18–20). Diet-related differences in gut microbiota have been described in older adults, and a Mediterranean diet intervention altered gut microbiome profiles while improving frailty-related health measures (21, 22, 34). Chronic inflammation is also closely linked to aging, cardiovascular disease and frailty, providing another plausible pathway through which fiber-rich dietary patterns could influence physical resilience (23).

Importantly, these findings do not isolate dietary fiber as an independent causal factor. At baseline, participants in the highest fiber-density quartile were less likely to smoke, were more physically active, reported better health and were more often women than those in the lowest quartile. Additional adjustment for broad diet quality attenuated the continuous-exposure odds ratio from 0.77 to 0.85. Fiber density may therefore index a broader constellation of dietary quality, health consciousness, socioeconomic resources and lifestyle behaviors. Multivariable adjustment, selection weighting, diet-pattern adjustment and *E*-values cannot eliminate residual confounding from imprecisely measured or unmeasured characteristics such as health literacy, food access and long-term dietary habits.

Fiber is also heterogeneous. Fibers from fruits, vegetables, legumes and whole grains differ in solubility, viscosity, fermentability, food matrix and accompanying micronutrients, and may not have equivalent biological effects. The HCNS total-fiber nutrient variable used here does not partition fiber grams by food source. Although food-context models additionally adjusted for whole grains, nuts, fish, sugar-sweetened beverages and selected plant-food markers, these models cannot attribute the association to a specific fiber source. This limits biological specificity and motivates future source-specific analyses.

The study has several strengths. HRS provides a well-characterized, nationally relevant longitudinal aging platform, and the analysis used baseline exclusion of prevalent gross motor limitation to focus on incident functional decline. The findings were tested across alternative exposure definitions, stricter outcome definitions, multiple imputation, complete-case selection weighting, health-selection restrictions, Fine-Gray competing-risk analysis, discrete-time hazard models, approximate Cox models and secondary functional outcomes. Risk ratios were prioritized because the outcome was common, and absolute-risk contrasts were included to improve interpretability (29, 30). *E*-value analyses were used as descriptive benchmarks for unmeasured confounding rather than as proof of causality (31).

Several limitations should temper interpretation. Diet was measured once using self-reported food-frequency data, which are vulnerable to measurement error and systematic reporting bias (35, 36); the exact HCNS adaptation has not been separately validated. The total-fiber measure also precluded source-specific inference. Functional outcomes were assessed at biennial waves, so exact onset dates were not observed; Cox and Fine-Gray models were therefore sensitivity analyses rather than the primary inferential framework. Covariate missingness was low and multiple-imputation estimates were stable, but imputation does not address measurement error or unmeasured confounding. Reverse causation also remains possible despite excluding prevalent gross motor limitation. Preclinical decline, fatigue, pain, dentition, appetite or reduced ability to shop and prepare food could have influenced baseline diet before the gross-motor count became non-zero. Excluding early events and restricting analyses to healthier participants reduced this concern but could not remove it.

The attenuated association among participants aged 75 years or older has several non-exclusive explanations. Their weighted gross-motor event rate was 55.4%, compared with 23.8% among those younger than 65 years and 37.3% among those aged 65–74 years. Survivor selection, greater exposure to competing mortality and the high baseline risk of limitation may compress relative contrasts. Accumulated or irreversible pathology may also leave less modifiable reserve, while one baseline dietary assessment may represent later follow-up less well at advanced ages. Moreover, evidence of interaction depended on effect scale: it survived false-discovery-rate adjustment in modified Poisson models but not in logistic models. The subgroup result should therefore not be interpreted as a discrete biological threshold or as evidence that fiber-rich diets have no relevance after age 75. Reporting and interpretation remain aligned with nutritional epidemiology guidance (37).

Taken together, the results identify fiber density as a prospective marker of lower functional-limitation risk, but the marker is embedded in a wider dietary and lifestyle pattern. The findings do not establish that increasing fiber intake will prevent gross motor limitation and do not define an intake target. Future studies should use repeated dietary assessments, quantify source-specific fiber, incorporate gut microbial metabolites and inflammatory biomarkers, and test whether dietary interventions preserve mobility before disability emerges.

## Conclusion

In this prospective cohort of middle-aged and older adults, higher dietary fiber density was consistently associated with lower subsequent risk of incident gross motor limitation. However, fiber density may reflect a wider dietary and lifestyle profile, and residual confounding and reverse causation cannot be excluded. The estimates should not be interpreted as an intervention effect or intake target. Confirmation requires repeated dietary assessment, source-specific analyses and intervention-based studies.

## Data Availability

The data analyzed in this study are available from the Health and Retirement Study (HRS), including the 2013 Health Care and Nutrition Study and the RAND HRS Longitudinal File. Access is subject to the HRS data-use registration and data-use agreement. Derived analytic code and result-level source files can be made available by the corresponding author upon reasonable request, subject to the data-use requirements.
