# Supplementary material for: Dietary fiber density and incident gross motor limitation among middle-aged and older adults: a prospective cohort analysis
Source: Front Nutr. 2026 Aug 28;13:1922715. doi: 10.3389/fnut.2026.1922715 (PMC13561844; doi:10.3389/fnut.2026.1922715)
Supplement: Supplementary file 1 [file Data_Sheet_1.PDF]

Supplementary Information

Dietary fiber density and incident gross motor limitation among middle-aged and older adults

Contents

- Supplementary Methods
- Supplementary Figures 1-5
- Supplementary Tables 1-11

Supplementary Methods

Study data and analytic sample

The analysis used the 2013 Health Care and Nutrition Study linked to RAND Health and Retirement Study longitudinal data. Baseline functional status was taken from HRS wave 11, dietary intake was assessed in the 2013 HCNS, and follow-up functional status was evaluated from waves 12 to 16. Participants were eligible if they were aged 50 years or older, had a positive HCNS nutrition weight, had non-missing dietary fiber density, had no gross motor limitation at baseline and had observed follow-up outcome status. Of 5,504 outcome-known eligible participants, 5,388 had complete Model 3 covariate data and comprised the primary complete-case cohort.

Dietary assessment and exposure definition

Diet was assessed using the mailed, self-administered 2013 HCNS semiquantitative food-frequency questionnaire, which was based on the Harvard food-frequency questionnaire. Respondents reported average past-year intake for 164 listed foods and beverages using item-specific portions and frequency categories and could report up to three additional foods. HRS converted responses to servings per day and derived 215 nutrient variables using Harvard nutrient composition tables. In the final Version 5.0 nutrient file, HRS excluded 38 participants with extremely sparse or inapplicable dietary data and imputed missing food-item responses using age, sex, race, education, body mass index and six sentinel foods; 97% of respondents completed at least 90% of food items. The parent Harvard questionnaire has demonstrated validity and reproducibility for foods and food groups, but the exact HCNS adaptation has not been separately validated. The primary exposure was fiber density, calculated as AOFIB_SUM divided by CALOR_SUM and expressed as grams per 1,000 kcal. Sensitivity analyses evaluated total fiber, log-transformed total fiber, energy-residual fiber, winsorized fiber density, removal of extreme energy or fiber-density values and a plausible-energy restriction.

Outcome definition

The primary outcome was incident gross motor limitation. It is a 0-5 count based on five any-difficulty indicators: walking one block, walking across a room, climbing one flight of stairs, getting in or out of bed and bathing. In this analysis, r11grossa defined baseline status, and r12grossa to r16grossa defined follow-up status. Negative or out-of-range values were treated as missing. Participants with r11grossa equal to zero entered the incident-risk set, and incident limitation was defined as a count of one or more in any follow-up wave.

Covariates and model sequence

Model 1 adjusted for age, sex, race or ethnicity, education and total energy intake. Model 2 additionally adjusted for body mass index, current smoking, alcohol use and weekly moderate or vigorous physical activity. Model 3, the primary model, additionally adjusted for hypertension, diabetes and self-rated health. Continuous covariates were standardized when used in regression models.

Statistical analyses

Survey-weighted logistic regression was used for the primary odds-ratio estimates. Because the outcome was common, modified Poisson models were fitted to estimate risk ratios for interpretation. Absolute-risk contrasts comparing the highest and lowest quartiles of dietary fiber density were estimated using augmented inverse probability weighting with bootstrap confidence intervals. Dose-response patterns were assessed with survey-weighted natural spline models. Interval-aware sensitivity analyses used discrete-time hazard models and approximate Cox models based on the first wave in which gross motor limitation was reported.

Missing-data sensitivity analysis

Per-variable missingness was calculated among 5,504 eligible participants with observed follow-up outcome status. Primary models used complete cases. Multiple imputation by chained equations used 20 imputations and 10 iterations; the imputation model included the outcome, fiber density, HCNS nutrition weight and all Model 3 covariates. Predictive mean matching was used for education, body mass index and self-rated health; logistic regression for current smoking and physical activity; and multinomial logistic regression for race or ethnicity. Exposure and outcome were not imputed. Survey-weighted logistic and modified Poisson estimates were pooled using Rubin's rules.

Competing-risk sensitivity analysis

HCNS-weighted Fine-Gray models treated first observed gross motor limitation as the event and death before observed limitation as the competing event. Participants with neither outcome were censored at their last observed limitation-free wave. Models used the fully adjusted Model 3 covariates and robust standard errors clustered by participant. Timing was assigned to biennial interview waves, so subdistribution hazard ratios were interpreted as approximate sensitivity estimates.

Robustness, heterogeneity and secondary outcomes

Robustness analyses examined alternative exposure definitions, stricter outcome definitions, diet-pattern adjustment, food-source adjustment, complete-case selection weighting, competing death, health-selection restrictions and reverse-causation restrictions. Subgroup analyses evaluated heterogeneity by age, sex, obesity, physical activity, diabetes, hypertension, self-rated health and major chronic disease. False-discovery-rate correction was applied to interaction tests. Secondary functional outcomes included incident ADL disability, IADL disability and mobility limitation.

Supplementary Figures and Legends

Supplementary Figure 1. Analytic sample flow

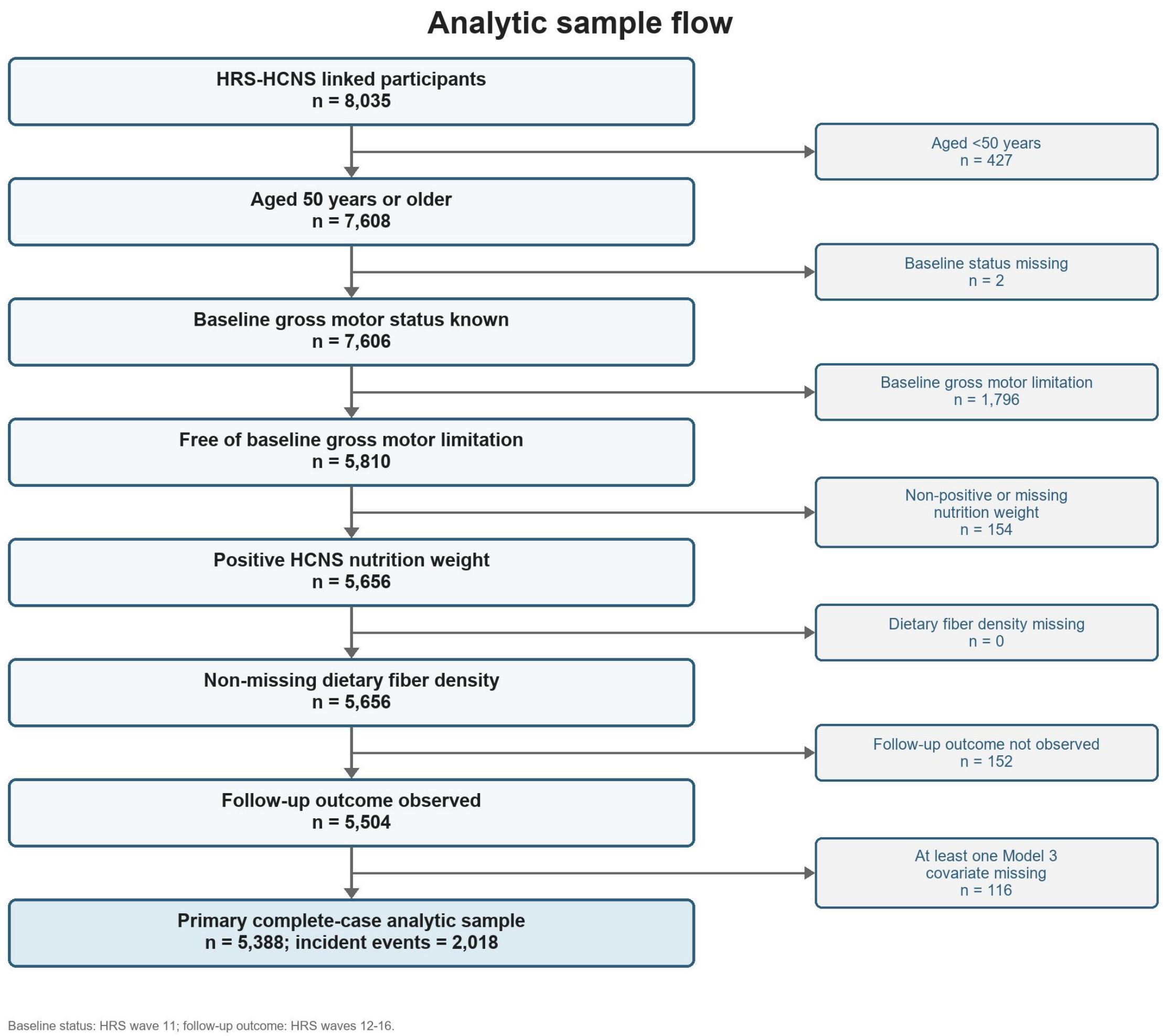

Supplementary Figure 1. Analytic sample flow

Supplementary Figure 2. Spline-based marginal risk curve

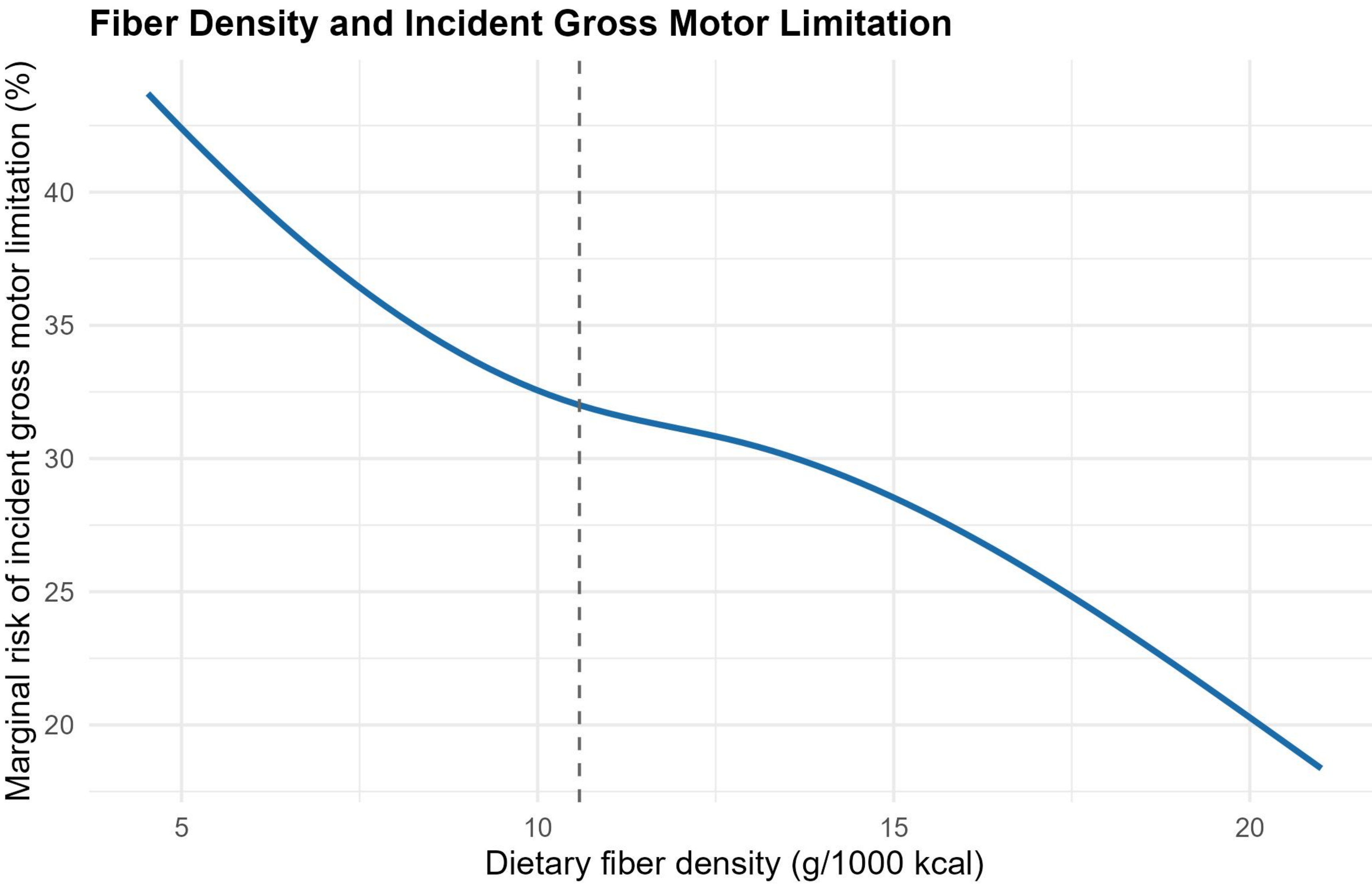

Supplementary Figure 2. Spline-based marginal risk curve

Supplementary Figure 3. Integrated robustness atlas, relative-effect scale

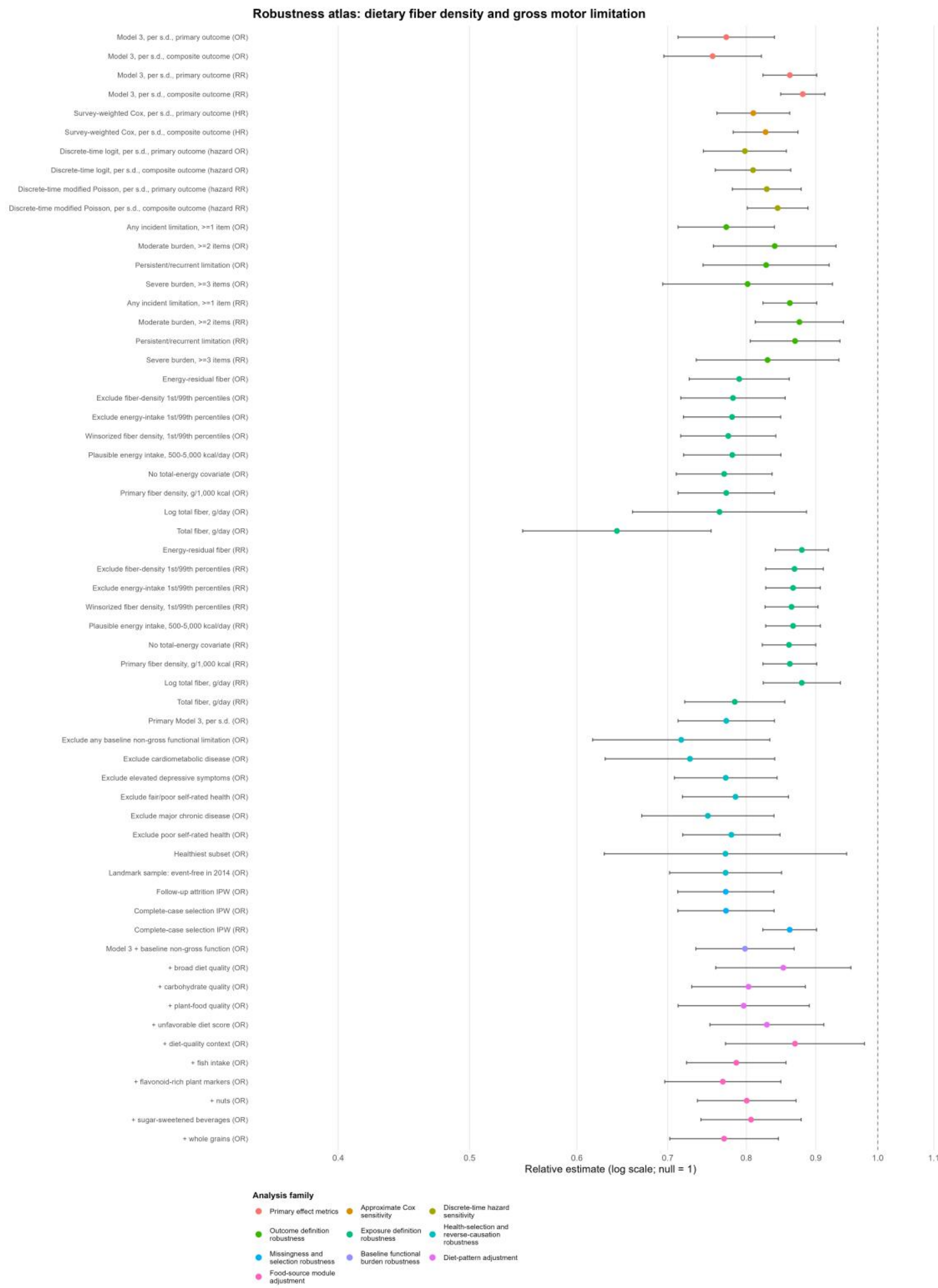

Supplementary Figure 3. Integrated robustness atlas, relative-effect scale

Supplementary Figure 4. Integrated robustness atlas, absolute-effect scale

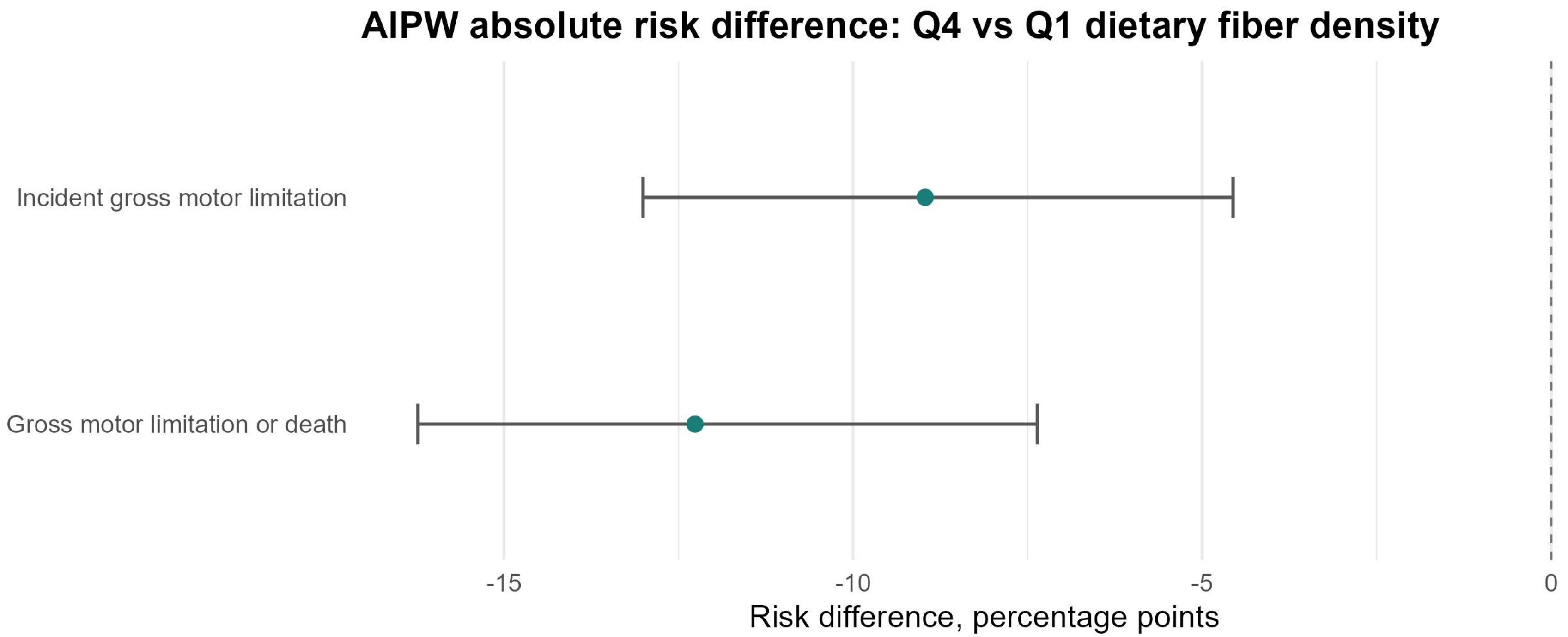

Supplementary Figure 4. Integrated robustness atlas, absolute-effect scale

Supplementary Figure 5. Subgroup robustness atlas

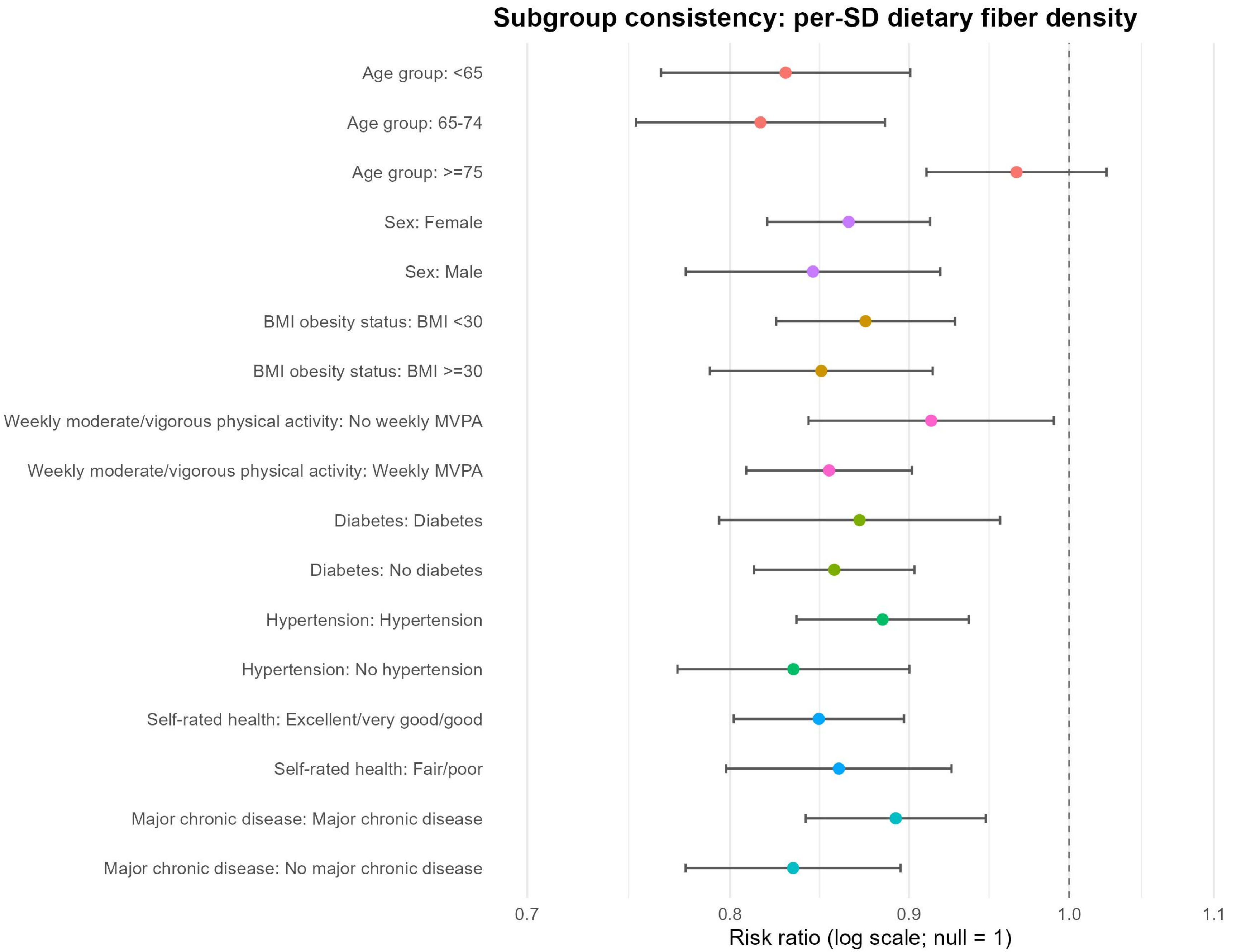

Supplementary Figure 5. Subgroup robustness atlas

Supplementary Tables

Supplementary Table 1. Analytic sample, missingness and exposure distribution

Panel A. Analytic sample flow

Analytic step	N remaining	Excluded at step
HRS-HCNS linked participants	8,035	0
Aged 50 years or older	7,608	427
Baseline gross motor limitation known	7,606	429
Free of baseline gross motor limitation at baseline	5,810	2,225
Positive HCNS nutrition weight	5,656	2,379
Non-missing dietary fiber density	5,656	2,379
Follow-up outcome known	5,504	2,531
Primary complete-case analytic sample	5,388	2,647

Panel B. Missingness among 5,504 outcome-known eligible participants

Missingness percentages use 5,504 participants as the denominator. Because some participants lacked more than one covariate, the number of participants excluded from the complete-case analysis (116) is smaller than the sum of the per-variable missing counts.

Variable	Missing, n	Missing, %
Incident gross motor limitation, waves 12-16	0	0
Dietary fiber density, g/1,000 kcal	0	0
Age	0	0
Female sex	0	0
Race or ethnicity	12	0.22
Education	24	0.44
Total energy intake, kcal/day	0	0
Body mass index	55	1.00
Current smoking	22	0.40
Any alcohol use	0	0
Weekly moderate or vigorous physical activity	3	0.05
Hypertension	0	0
Diabetes	0	0
Self-rated health	4	0.07

Panel C. Exposure distribution

Exposure	N	Weighted mean (s.d.)	P1	P25	P50	P75	P99
Fiber density, g/1,000 kcal	5,656	10.78 (3.42)	4.52	8.59	10.57	12.87	20.83
Total fiber, g/day	5,656	19.62 (11.26)	4.47	12.50	17.73	24.62	60.51
Total energy, kcal/day	5,656	1,850.2 (973.5)	524.8	1,256.1	1,677.3	2,227.0	5,032.2

Supplementary Table 2. Alternative exposure-definition sensitivity analyses

Panel A. Alternative fiber exposure definitions

Exposure definition	Restriction	Model	Measure	N	Events	Weighted event rate, %	Estimate (95% CI)	*P*
Fiber density, g/1,000 kcal		Logistic	Odds ratio	5,388	2,018	32.7	0.77 (0.71-0.84)	<0.001
Fiber density, g/1,000 kcal		Modified Poisson	Risk ratio	5,388	2,018	32.7	0.86 (0.82-0.90)	<0.001
Fiber density without total-energy covariate		Logistic	Odds ratio	5,388	2,018	32.7	0.77 (0.71-0.84)	<0.001
Fiber density without total-energy covariate		Modified Poisson	Risk ratio	5,388	2,018	32.7	0.86 (0.82-0.90)	<0.001
Total fiber, g/day		Logistic	Odds ratio	5,388	2,018	32.7	0.64 (0.55-0.75)	<0.001
Total fiber, g/day		Modified Poisson	Risk ratio	5,388	2,018	32.7	0.78 (0.72-0.85)	<0.001
Log total fiber, g/day		Logistic	Odds ratio	5,388	2,018	32.7	0.76 (0.66-0.89)	<0.001
Log total fiber, g/day		Modified Poisson	Risk ratio	5,388	2,018	32.7	0.88 (0.82-0.94)	<0.001
Energy-residual fiber		Logistic	Odds ratio	5,388	2,018	32.7	0.79 (0.73-0.86)	<0.001
Energy-residual fiber		Modified Poisson	Risk ratio	5,388	2,018	32.7	0.88 (0.84-0.92)	<0.001
Fiber density winsorized at 1st/99th percentile		Logistic	Odds ratio	5,388	2,018	32.7	0.78 (0.72-0.84)	<0.001
Fiber density winsorized at 1st/99th percentile		Modified Poisson	Risk ratio	5,388	2,018	32.7	0.86 (0.83-0.90)	<0.001
Fiber density excluding energy 1st/99th percentile	energy 1st-99th percentile	Logistic	Odds ratio	5,282	1,970	32.5	0.78 (0.72-0.85)	<0.001
Fiber density excluding energy 1st/99th percentile	energy 1st-99th percentile	Modified Poisson	Risk ratio	5,282	1,970	32.5	0.87 (0.83-0.91)	<0.001
Fiber density excluding density 1st/99th percentile	density 1st-99th percentile	Logistic	Odds ratio	5,277	1,983	32.8	0.78 (0.72-0.85)	<0.001
Fiber density excluding density 1st/99th percentile	density 1st-99th percentile	Modified Poisson	Risk ratio	5,277	1,983	32.8	0.87 (0.83-0.91)	<0.001
Fiber density with energy 500-5000 kcal/day	energy 500-5,000 kcal/day	Logistic	Odds ratio	5,287	1,970	32.5	0.78 (0.72-0.85)	<0.001
Fiber density with energy 500-5000 kcal/day	energy 500-5,000 kcal/day	Modified Poisson	Risk ratio	5,287	1,970	32.5	0.87 (0.83-0.91)	<0.001

Supplementary Table 3. Alternative outcome-definition sensitivity analyses

Panel A. Alternative gross motor limitation definitions

Outcome definition	Contrast	Model	Measure	N	Events	Weighted event rate, %	Estimate (95% CI)	P
Any incident gross motor limitation (>=1 item in any wave)	per s.d.	Logistic	Odds ratio	5,388	2,018	32.7	0.77 (0.71-0.84)	<0.001
Any incident gross motor limitation (>=1 item in any wave)	Q4 vs Q1	Logistic	Odds ratio	5,388	2,018	32.7	0.55 (0.44-0.68)	<0.001
Any incident gross motor limitation (>=1 item in any wave)	per s.d.	Modified Poisson	Risk ratio	5,388	2,018	32.7	0.86 (0.82-0.90)	<0.001
Any incident gross motor limitation (>=1 item in any wave)	Q4 vs Q1	Modified Poisson	Risk ratio	5,388	2,018	32.7	0.72 (0.64-0.81)	<0.001
Moderate burden (>=2 items in any wave)	per s.d.	Logistic	Odds ratio	5,388	1,068	16.6	0.84 (0.76-0.93)	<0.001
Moderate burden (>=2 items in any wave)	Q4 vs Q1	Logistic	Odds ratio	5,388	1,068	16.6	0.70 (0.53-0.91)	0.009
Moderate burden (>=2 items in any wave)	per s.d.	Modified Poisson	Risk ratio	5,388	1,068	16.6	0.88 (0.81-0.94)	<0.001
Moderate burden (>=2 items in any wave)	Q4 vs Q1	Modified Poisson	Risk ratio	5,388	1,068	16.6	0.77 (0.64-0.93)	0.006
Severe burden (>=3 items in any wave)	per s.d.	Logistic	Odds ratio	5,388	548	8.1	0.80 (0.69-0.93)	0.003
Severe burden (>=3 items in any wave)	Q4 vs Q1	Logistic	Odds ratio	5,388	548	8.1	0.66 (0.47-0.94)	0.02
Severe burden (>=3 items in any wave)	per s.d.	Modified Poisson	Risk ratio	5,388	548	8.1	0.83 (0.73-0.94)	0.002
Severe burden (>=3 items in any wave)	Q4 vs Q1	Modified Poisson	Risk ratio	5,388	548	8.1	0.71 (0.54-0.95)	0.021
Persistent/recurrent limitation (>=1 item in >=2 follow-up waves)	per s.d.	Logistic	Odds ratio	4,945	1,011	16.9	0.83 (0.74-0.92)	<0.001
Persistent/recurrent limitation (>=1 item in >=2 follow-up waves)	Q4 vs Q1	Logistic	Odds ratio	4,945	1,011	16.9	0.60 (0.45-0.80)	<0.001
Persistent/recurrent limitation (>=1 item in >=2 follow-up waves)	per s.d.	Modified Poisson	Risk ratio	4,945	1,011	16.9	0.87 (0.81-0.94)	<0.001
Persistent/recurrent limitation (>=1 item in >=2 follow-up waves)	Q4 vs Q1	Modified Poisson	Risk ratio	4,945	1,011	16.9	0.69 (0.57-0.85)	<0.001

Supplementary Table 4. Nested primary models, quartile contrasts and core effect metrics

Panel A. Nested continuous-exposure logistic models

Model	N	Events	OR (95% CI)	P
Model 1	5,468	2,048	0.70 (0.65-0.75)	<0.001
Model 2	5,392	2,019	0.76 (0.71-0.83)	<0.001
Model 3	5,388	2,018	0.77 (0.71-0.84)	<0.001

Panel B. Fully adjusted quartile logistic model

Contrast	N	Events	OR (95% CI)	P
Q2 versus Q1	5,388	2,018	0.73 (0.59-0.91)	0.004
Q3 versus Q1	5,388	2,018	0.70 (0.57-0.87)	0.001
Q4 versus Q1	5,388	2,018	0.55 (0.44-0.69)	<0.001
Quartile median trend	5,388	2,018	0.81 (0.75-0.88)	<0.001

Panel C. Core effect metrics for incident gross motor limitation

Model	Measure	Contrast	N	Events	Weighted event rate, %	Estimate (95% CI)	P
Logistic, per s.d.	Odds ratio	per s.d.	5,388	2,018	32.7	0.77 (0.71-0.84)	<0.001
Logistic, Q4 versus Q1	Odds ratio	Q4 vs Q1	5,388	2,018	32.7	0.55 (0.44-0.68)	<0.001
Modified Poisson, per s.d.	Risk ratio	per s.d.	5,388	2,018	32.7	0.86 (0.82-0.90)	<0.001
Modified Poisson, Q4 versus Q1	Risk ratio	Q4 vs Q1	5,388	2,018	32.7	0.72 (0.64-0.81)	<0.001

Supplementary Table 5. Interval-aware, approximate Cox and competing-event sensitivity analyses

Panel A. Discrete-time hazard models

Analysis	Family	Measure	Contrast	Person-periods	Participants	Events	Estimate (95% CI)	P
Gross motor	Logistic	Hazard odds ratio	per s.d.	17,623	5,313	1,923	0.80 (0.74-0.86)	<0.001
Gross motor	Logistic	Hazard odds ratio	Q4 vs Q1	17,623	5,313	1,923	0.58 (0.48-0.70)	<0.001
Gross motor	modified Poisson	Hazard risk ratio	per s.d.	17,623	5,313	1,923	0.83 (0.78-0.88)	<0.001
Gross motor	modified Poisson	Hazard risk ratio	Q4 vs Q1	17,623	5,313	1,923	0.64 (0.55-0.74)	<0.001
Gross motor or death	Logistic	Hazard odds ratio	per s.d.	18,083	5,366	2,383	0.81 (0.76-0.86)	<0.001
Gross motor or death	Logistic	Hazard odds ratio	Q4 vs Q1	18,083	5,366	2,383	0.57 (0.48-0.67)	<0.001
Gross motor or death	modified Poisson	Hazard risk ratio	per s.d.	18,083	5,366	2,383	0.84 (0.80-0.89)	<0.001
Gross motor or death	modified Poisson	Hazard risk ratio	Q4 vs Q1	18,083	5,366	2,383	0.64 (0.56-0.73)	<0.001

Panel B. Approximate Cox models

Analysis set	Contrast	N	Events	Censored	Median follow-up, years	HR (95% CI)	P
Main binary complete case	per s.d.	5,388	2,018	3,370	8	0.81 (0.76-0.86)	<0.001
Main binary complete case	Q4 vs Q1	5,388	2,018	3,370	8	0.60 (0.51-0.70)	<0.001
Main binary complete case	per s.d.	5,388	2,532	2,856	8	0.83 (0.78-0.87)	<0.001
Main binary complete case	Q4 vs Q1	5,388	2,532	2,856	8	0.60 (0.52-0.69)	<0.001
Time available	per s.d.	5,450	2,018	3,432	8	0.81 (0.76-0.86)	<0.001
Time available	Q4 vs Q1	5,450	2,018	3,432	8	0.60 (0.51-0.70)	<0.001
Time available	per s.d.	5,450	2,594	2,856	8	0.83 (0.78-0.87)	<0.001
Time available	Q4 vs Q1	5,450	2,594	2,856	8	0.61 (0.53-0.70)	<0.001

Panel C. Composite endpoint of incident gross motor limitation or death

Model	Measure	Contrast	N	Events	Weighted event rate, %	Estimate (95% CI)	P
Logistic, per s.d.	Odds ratio	per s.d.	5,450	2,594	41.2	0.76 (0.70-0.82)	<0.001
Logistic, Q4 versus Q1	Odds ratio	Q4 vs Q1	5,450	2,594	41.2	0.48 (0.38-0.60)	<0.001
Modified Poisson, per s.d.	Risk ratio	per s.d.	5,450	2,594	41.2	0.88 (0.85-0.91)	<0.001
Modified Poisson, Q4 versus Q1	Risk ratio	Q4 vs Q1	5,450	2,594	41.2	0.73 (0.67-0.81)	<0.001

Supplementary Table 6. Diet-pattern and food-source adjustment analyses

Panel A. Fiber estimates after broader diet-pattern adjustment

Diet module	N	Events	Fiber OR (95% CI)	P	Attenuation from base OR, %
Carbohydrate quality	5,388	2,018	0.80 (0.73-0.88)	<0.001	14.7
Plant food quality	5,388	2,018	0.80 (0.71-0.89)	<0.001	11.5
Unfavorable diet	5,388	2,018	0.83 (0.75-0.91)	<0.001	26.8
Broad diet quality	5,388	2,018	0.85 (0.76-0.96)	0.006	37.7

Panel B. Fiber estimates after food-source module adjustment

Food-source module	N	Events	Fiber OR (95% CI)	P	Attenuation from base OR, %
Whole grain	5,388	2,018	0.77 (0.70-0.84)	<0.001	-1.4
Nuts	5,388	2,018	0.80 (0.74-0.87)	<0.001	13.5
Fish	5,388	2,018	0.79 (0.72-0.86)	<0.001	6.6
Sugar sweetened beverages	5,388	2,018	0.81 (0.74-0.88)	<0.001	16.4
Flavonoid rich plant markers	5,388	2,018	0.77 (0.70-0.85)	<0.001	-2.3
Diet quality context	5,388	2,018	0.87 (0.77-0.98)	0.02	45.4

Supplementary Table 7. Complete-case selection, health-selection and reverse-causation analyses

Panel A. Complete-case selection diagnostics

Eligible N	Complete-case N	Complete-case, %	Pr(complete), min	Pr(complete), p1	Pr(complete), median	Pr(complete), p99	Pr(complete), max
5,504	5,388	97.9	0.820	0.922	0.985	0.990	0.990

Panel B. Complete-case selection IPW models

Analysis	Weight	Model	Measure	Contrast	N	Events	Estimate (95% CI)	P
Standard complete-case weighting	Weight	Logistic	Odds ratio	per s.d.	5,388	2,018	0.77 (0.71-0.84)	<0.001
Standard complete-case weighting	Weight	Logistic	Odds ratio	Q4 vs Q1	5,388	2,018	0.55 (0.44-0.68)	<0.001
Standard complete-case weighting	Weight	Modified Poisson	Risk ratio	per s.d.	5,388	2,018	0.86 (0.82-0.90)	<0.001
Standard complete-case weighting	Weight	Modified Poisson	Risk ratio	Q4 vs Q1	5,388	2,018	0.72 (0.64-0.81)	<0.001
Complete-case selection IPW	Selection-IPW weight	Logistic	Odds ratio	per s.d.	5,388	2,018	0.77 (0.71-0.84)	<0.001
Complete-case selection IPW	Selection-IPW weight	Logistic	Odds ratio	Q4 vs Q1	5,388	2,018	0.55 (0.44-0.68)	<0.001
Complete-case selection IPW	Selection-IPW weight	Modified Poisson	Risk ratio	per s.d.	5,388	2,018	0.86 (0.82-0.90)	<0.001
Complete-case selection IPW	Selection-IPW weight	Modified Poisson	Risk ratio	Q4 vs Q1	5,388	2,018	0.72 (0.64-0.81)	<0.001

Panel C. Health-selection and reverse-causation restrictions

Analysis	Contrast	N	Events	Weighted event rate, %	OR (95% CI)	P
Base Model 3	per s.d.	5,388	2,018	32.7	0.77 (0.71-0.84)	<0.001
Base Model 3	Q4 vs Q1	5,388	2,018	32.7	0.55 (0.44-0.68)	<0.001
Exclude fair or poor self-rated health	per s.d.	4,600	1,526	27.9	0.79 (0.72-0.86)	<0.001
Exclude fair or poor self-rated health	Q4 vs Q1	4,600	1,526	27.9	0.60 (0.47-0.76)	<0.001
Exclude poor self-rated health	per s.d.	5,295	1,948	31.8	0.78 (0.72-0.85)	<0.001
Exclude poor self-rated health	Q4 vs Q1	5,295	1,948	31.8	0.56 (0.45-0.70)	<0.001
Exclude major chronic disease	per s.d.	2,938	868	25.1	0.75 (0.67-0.84)	<0.001
Exclude major chronic disease	Q4 vs Q1	2,938	868	25.1	0.50 (0.37-0.68)	<0.001
Exclude cardiometabolic conditions	per s.d.	1,898	486	21.6	0.73 (0.63-0.84)	<0.001
Exclude cardiometabolic conditions	Q4 vs Q1	1,898	486	21.6	0.47 (0.32-0.71)	<0.001
Exclude elevated depressive symptoms	per s.d.	4,857	1,730	30.5	0.77 (0.71-0.84)	<0.001
Exclude elevated depressive symptoms	Q4 vs Q1	4,857	1,730	30.5	0.55 (0.43-0.69)	<0.001
Exclude any baseline non-gross function burden	per s.d.	2,399	452	16.2	0.72 (0.62-0.83)	<0.001
Exclude any baseline non-gross function burden	Q4 vs Q1	2,399	452	16.2	0.46 (0.30-0.71)	<0.001
Healthiest subset	per s.d.	1,422	183	10.6	0.77 (0.63-0.95)	0.014
Healthiest subset	Q4 vs Q1	1,422	183	10.6	0.58 (0.33-1.02)	0.058
Landmark 2014 event free	per s.d.	4,355	1,345	26.9	0.77 (0.70-0.85)	<0.001
Landmark 2014 event free	Q4 vs Q1	4,355	1,345	26.9	0.53 (0.41-0.69)	<0.001

Supplementary Table 8. Exploratory subgroup heterogeneity and sex-stratified analyses

Panel A. Interaction tests

Subgroup	Model	Measure	N	Events	Interaction P	FDR-adjusted P
Age group	Logistic	Odds ratio	5,388	2,018	0.008	0.063
Age group	Modified Poisson	Risk ratio	5,388	2,018	<0.001	<0.001
Sex	Logistic	Odds ratio	5,388	2,018	0.559	0.807
Sex	Modified Poisson	Risk ratio	5,388	2,018	0.281	0.563
BMI obesity status	Logistic	Odds ratio	5,388	2,018	0.807	0.807
BMI obesity status	Modified Poisson	Risk ratio	5,388	2,018	0.925	0.925
Weekly moderate/vigorous physical activity	Logistic	Odds ratio	5,388	2,018	0.765	0.807
Weekly moderate/vigorous physical activity	Modified Poisson	Risk ratio	5,388	2,018	0.824	0.925
Diabetes	Logistic	Odds ratio	5,388	2,018	0.738	0.807
Diabetes	Modified Poisson	Risk ratio	5,388	2,018	0.421	0.674
Hypertension	Logistic	Odds ratio	5,388	2,018	0.368	0.736
Hypertension	Modified Poisson	Risk ratio	5,388	2,018	0.099	0.264
Self-rated health	Logistic	Odds ratio	5,388	2,018	0.277	0.736
Self-rated health	Modified Poisson	Risk ratio	5,388	2,018	0.772	0.925
Major chronic disease	Logistic	Odds ratio	5,388	2,018	0.305	0.736
Major chronic disease	Modified Poisson	Risk ratio	5,388	2,018	0.065	0.261

Panel B. Stratum-specific effect estimates

Subgroup	Level	Model	Measure	N	Events	Weighted event rate, %	Estimate (95% CI)	P
Age group	<65	Logistic	Odds ratio	2,485	666	23.8	0.75 (0.66-0.85)	<0.001
Age group	<65	Modified Poisson	Risk ratio	2,485	666	23.8	0.83 (0.76-0.90)	<0.001
Age group	65-74	Logistic	Odds ratio	1,576	622	37.3	0.70 (0.61-0.82)	<0.001
Age group	65-74	Modified Poisson	Risk ratio	1,576	622	37.3	0.82 (0.75-0.89)	<0.001
Age group	>=75	Logistic	Odds ratio	1,327	730	55.4	0.93 (0.81-1.06)	0.288
Age group	>=75	Modified Poisson	Risk ratio	1,327	730	55.4	0.97 (0.91-1.02)	0.253
Sex	Female	Logistic	Odds ratio	3,010	1,249	36.9	0.78 (0.70-0.86)	<0.001
Sex	Female	Modified Poisson	Risk ratio	3,010	1,249	36.9	0.86 (0.82-0.91)	<0.001
Sex	Male	Logistic	Odds ratio	2,378	769	28.1	0.77 (0.67-0.88)	<0.001
Sex	Male	Modified Poisson	Risk ratio	2,378	769	28.1	0.84 (0.78-0.92)	<0.001
BMI obesity status	BMI <30	Logistic	Odds ratio	3,668	1,227	28.6	0.80 (0.73-0.88)	<0.001
BMI obesity status	BMI <30	Modified Poisson	Risk ratio	3,668	1,227	28.6	0.87 (0.82-0.93)	<0.001
BMI obesity status	BMI >=30	Logistic	Odds ratio	1,720	791	41.4	0.72 (0.62-0.84)	<0.001
BMI obesity status	BMI >=30	Modified	Risk ratio	1,720	791	41.4	0.85 (0.79-0.91)	<0.001

Subgroup	Level	Model	Measure	N	Events	Weighted event rate, %	Estimate (95% CI)	P
		Poisson						
Weekly moderate/vigorous physical activity	No weekly MVPA	Logistic	Odds ratio	1,101	593	52.4	0.82 (0.68-0.98)	0.031
Weekly moderate/vigorous physical activity	No weekly MVPA	Modified Poisson	Risk ratio	1,101	593	52.4	0.91 (0.84-0.99)	0.028
Weekly moderate/vigorous physical activity	Weekly MVPA	Logistic	Odds ratio	4,287	1,425	27.9	0.77 (0.70-0.84)	<0.001
Weekly moderate/vigorous physical activity	Weekly MVPA	Modified Poisson	Risk ratio	4,287	1,425	27.9	0.85 (0.81-0.90)	<0.001
Diabetes	Diabetes	Logistic	Odds ratio	1,009	498	45.9	0.77 (0.64-0.93)	0.007
Diabetes	Diabetes	Modified Poisson	Risk ratio	1,009	498	45.9	0.87 (0.79-0.96)	0.004
Diabetes	No diabetes	Logistic	Odds ratio	4,379	1,520	30.0	0.77 (0.70-0.84)	<0.001
Diabetes	No diabetes	Modified Poisson	Risk ratio	4,379	1,520	30.0	0.86 (0.81-0.90)	<0.001
Hypertension	Hypertension	Logistic	Odds ratio	2,966	1,320	41.0	0.79 (0.71-0.89)	<0.001
Hypertension	Hypertension	Modified Poisson	Risk ratio	2,966	1,320	41.0	0.88 (0.84-0.94)	<0.001
Hypertension	No hypertension	Logistic	Odds ratio	2,422	698	24.3	0.75 (0.66-0.84)	<0.001
Hypertension	No hypertension	Modified Poisson	Risk ratio	2,422	698	24.3	0.83 (0.77-0.90)	<0.001
Self-rated health	Excellent/very good/good	Logistic	Odds ratio	4,600	1,526	27.9	0.77 (0.70-0.84)	<0.001
Self-rated health	Excellent/very good/good	Modified Poisson	Risk ratio	4,600	1,526	27.9	0.85 (0.80-0.90)	<0.001
Self-rated health	Fair/poor	Logistic	Odds ratio	788	492	61.6	0.66 (0.54-0.80)	<0.001
Self-rated health	Fair/poor	Modified Poisson	Risk ratio	788	492	61.6	0.86 (0.80-0.93)	<0.001
Major chronic disease	Major chronic disease	Logistic	Odds ratio	2,450	1,150	43.4	0.80 (0.71-0.90)	<0.001
Major chronic disease	Major chronic disease	Modified Poisson	Risk ratio	2,450	1,150	43.4	0.89 (0.84-0.95)	<0.001
Major chronic disease	No major chronic disease	Logistic	Odds ratio	2,938	868	25.1	0.75 (0.67-0.84)	<0.001
Major chronic disease	No major chronic disease	Modified Poisson	Risk ratio	2,938	868	25.1	0.83 (0.78-0.89)	<0.001

Panel C. Subgroup-specific event rates

Subgroup	Level	N	Events	Crude event rate, %	Weighted event rate, %
Age group	<65	2,485	666	26.8	23.8
Age group	65-74	1,576	622	39.5	37.3
Age group	>=75	1,327	730	55.0	55.4
Sex	Female	3,010	1,249	41.5	36.9
Sex	Male	2,378	769	32.3	28.1

Subgroup	Level	N	Events	Crude event rate, %	Weighted event rate, %
BMI obesity status	BMI <30	3,668	1,227	33.5	28.6
BMI obesity status	BMI >=30	1,720	791	46.0	41.4
Weekly moderate/vigorous physical activity	No weekly MVPA	1,101	593	53.9	52.4
Weekly moderate/vigorous physical activity	Weekly MVPA	4,287	1,425	33.2	27.9
Diabetes	Diabetes	1,009	498	49.4	45.9
Diabetes	No diabetes	4,379	1,520	34.7	30.0
Hypertension	Hypertension	2,966	1,320	44.5	41.0
Hypertension	No hypertension	2,422	698	28.8	24.3
Self-rated health	Excellent/very good/good	4,600	1,526	33.2	27.9
Self-rated health	Fair/poor	788	492	62.4	61.6
Major chronic disease	Major chronic disease	2,450	1,150	46.9	43.4
Major chronic disease	No major chronic disease	2,938	868	29.5	25.1

Supplementary Table 9. Unmeasured-confounding benchmark analyses

Panel A. E-value benchmarks for selected estimates

Source	N	Events	OR (95% CI)	P	E-value	E-value CI limit
Main Model 3 weighted	5,388	2,018	0.77 (0.71-0.84)	<0.001	1.90	1.67
Plausible energy intake, 500-5,000 kcal/day	5,287	1,970	0.78 (0.72-0.85)	<0.001	1.87	1.63
Unweighted Model 3	5,388	2,018	0.83 (0.77-0.89)	<0.001	1.71	1.51
Additional baseline CVD adjusted	5,388	2,018	0.78 (0.72-0.84)	<0.001	1.90	1.66
Lagged baseline at wave 12, follow-up waves 13-16	4,731	1,607	0.78 (0.71-0.85)	<0.001	1.89	1.63
M3 plus baseline non-gross function burden	5,388	2,018	0.80 (0.73-0.87)	<0.001	1.82	1.57
M3 with follow-up attrition IPW	5,388	2,018	0.77 (0.71-0.84)	<0.001	1.91	1.67

Supplementary Table 10. Secondary functional-outcome analyses

Panel A. Incident ADL, IADL and mobility outcomes

Outcome	Definition	N	Events	OR per s.d. fiber density (95% CI)	P
Incident ADL disability	No ADL disability at wave 11 to any ADL disability during waves 12-16	5,938	1,653	0.89 (0.82-0.97)	0.006
Incident IADL disability	No IADL disability at wave 11 to any IADL disability during waves 12-16	6,100	1,530	0.88 (0.81-0.96)	0.003
Incident mobility limitation	No mobility limitation at wave 11 to any mobility limitation during waves 12-16	3,664	2,017	0.87 (0.79-0.96)	0.004

Models use the same fully adjusted covariate structure as the primary Model 3. Estimates are from the formal survey-weighted secondary-outcome models; event counts are drawn from the matched weighted outcome-screening output because the formal secondary-outcome summary stores event counts as missing.

Supplementary Table 11. Multiple-imputation and Fine-Gray competing-risk sensitivity analyses

Panel A. Complete-case and multiple-imputation estimates

Analysis	Contrast	Measure	N	Events	Estimate (95% CI)	P
Complete case	per 1-SD	OR	5,388	2,018	0.77 (0.71-0.84)	<0.001
Complete case	Q4 vs Q1	OR	5,388	2,018	0.55 (0.44-0.68)	<0.001
Complete case	per 1-SD	RR	5,388	2,018	0.86 (0.82-0.90)	<0.001
Complete case	Q4 vs Q1	RR	5,388	2,018	0.72 (0.64-0.81)	<0.001
Multiple imputation	per 1-SD	OR	5,504	2,057	0.77 (0.71-0.84)	<0.001
Multiple imputation	Q4 vs Q1	OR	5,504	2,057	0.54 (0.44-0.67)	<0.001
Multiple imputation	per 1-SD	RR	5,504	2,057	0.86 (0.82-0.90)	<0.001
Multiple imputation	Q4 vs Q1	RR	5,504	2,057	0.72 (0.64-0.81)	<0.001

Multiple-imputation estimates were pooled across 20 chained-equation datasets using Rubin's rules. OR, odds ratio; RR, risk ratio; SD, standard deviation.

Panel B. Fine-Gray competing-risk estimates

Contrast	N	Gross motor events	Competing deaths	Censored	sHR (95% CI)	P
per 1-SD	5,388	2,018	514	2,856	0.82 (0.77-0.87)	<0.001
Q4 vs Q1	5,388	2,018	514	2,856	0.62 (0.53-0.73)	<0.001

Fine-Gray models used HCNS nutrition weights and the fully adjusted Model 3 covariates, with robust standard errors clustered by participant. Death before observed gross motor limitation was treated as the competing event. Event timing was assigned to biennial waves. sHR, subdistribution hazard ratio.
